# Construction and application of a transitional health management program for children with Hirschsprung disease undergoing colonic irrigation based on the intervention mapping framework

**DOI:** 10.3389/fped.2026.1962532

**Published:** 2026-10-28

**Authors:** Yuanyuan Fang, Yulan Kang, Pingping Qiu

**Affiliations:** 1 Fujian Children’s Hospital (Fujian Branch of Shanghai Children’s Medical Center), College of Clinical Medicine for Obstetrics & Gynecology and Pediatrics, Fujian Medical University, Fuzhou, Fujian Province, China; 2 School of Nursing, Fujian Medical University, Fuzhou, Fujian Province, China

**Keywords:** Hirschsprung disease, intervention mapping theory, multidisciplinary care, retrograde enema, transitional care

## Abstract

**Objective:**

To develop an Intervention Mapping (IM) theory-based transitional health management program for children with Hirschsprung disease (HD) undergoing retrograde enema and evaluate its effectiveness.

**Methods:**

Guided by IM theory, the intervention was developed through semi-structured interviews, literature review, expert consultation, and a pilot study. From April 2024 to June 2025, 94 children with HD and their primary caregivers were recruited from a tertiary pediatric hospital in Fujian Province and randomly assigned to an intervention group (*n* = 47) or control group (*n* = 47). The intervention group received multidisciplinary transitional health management based on IM theory, while the control group received routine care. Bowel preparation, enema-related complications, nutritional and growth outcomes, and caregivers’ caregiving ability, anxiety, and depression were assessed.

**Results:**

Eighty-three participants completed follow-up (intervention, *n* = 42; control, *n* = 41). Compared with the control group, the intervention group had better bowel preparation, lower incidences of enterocolitis and overall complications, and more favorable preoperative nutritional and growth outcomes. Caregivers in the intervention group showed significantly greater caregiving ability and greater improvements in anxiety and depression at 1 week and 1 month after discharge (all *P* < 0.05). No significant differences were observed in enema-related dermatitis or intestinal obstruction (*P* > 0.05). Repeated-measures analyses demonstrated significant group-by-time interactions for caregiving ability, anxiety, and depression (*P* < 0.05).

**Conclusion:**

The IM theory-based transitional health management program is feasible and effective in improving clinical and caregiver-related outcomes in children with HD receiving retrograde enema and their families.

## 1 Introduction

Hirschsprung disease (HD) is a common congenital gastrointestinal malformation, affecting approximately 1 in 5,000 live births and accounting for about 20% of neonatal intestinal obstruction cases. Typical manifestations include delayed meconium passage, refractory constipation, abdominal distension, and vomiting (1, 2). Delayed treatment may lead to intestinal obstruction, perforation, sepsis, and even death (3, 4). Surgery is the definitive treatment (5), while retrograde enema is an essential component of preoperative management that facilitates fecal evacuation and reduces complications (6). Effective bowel decompression followed by surgery at approximately 3 months of age has been associated with improved outcomes (7, 8). Consequently, children with HD may require several months of home-based retrograde enema before surgery, with primary caregivers assuming responsibility for the procedure and daily care.

However, retrograde enema is an invasive procedure, and caregivers often have limited knowledge and skills while also managing disease monitoring and nutritional care. These challenges increase caregiving burden and procedural risks and may contribute to caregiver anxiety and depression, potentially affecting children's recovery and family quality of life (9–11). Existing HD nursing research has focused mainly on postoperative care, whereas systematic interventions targeting preoperative home-based retrograde enema remain limited (12, 13).

Intervention Mapping (IM) theory provides a systematic framework for developing evidence-based health interventions and has been widely applied in chronic disease management (14–17). Therefore, this study integrated IM theory, multidisciplinary collaboration, and mobile health technology to develop a transitional health management program for children with HD receiving home-based retrograde enema.

## 2 Methods

### 2.1 Study design and participants

This randomized controlled trial followed the Consolidated Standards of Reporting Trials (CONSORT) guidelines (18). Children with HD and their primary caregivers were recruited from the Department of General and Neonatal Surgery of a tertiary pediatric hospital in Fujian Province between April 2024 and June 2025. The study was approved by the institutional ethics committee (No. 2022ETKLR08032), and written informed consent was obtained.

Children were eligible if they: (1) were aged 0–2 years and newly diagnosed with HD according to established criteria (19); (2) required conservative retrograde enema before surgery; and (3) were full-term, first-born infants delivered vaginally with an Apgar score ≥7. Children with life-threatening organic diseases, major congenital malformations, or conditions affecting treatment were excluded.

Caregivers were eligible if they: (1) were aged <65 years; (2) could independently perform three retrograde enemas before discharge; (3) were primarily responsible for enema administration and daily care; and (4) could use WeChat and complete online questionnaires independently. Caregivers were excluded if they employed professional caregivers, had a history of psychiatric or psychological disorders, or had severe physical illness.

HD was classified according to the subtypes defined in the Chinese Expert Consensus on Hirschsprung Disease: short-segment type, with aganglionosis confined to the mid and distal rectum; common type, with aganglionosis extending proximally from the anus to the proximal rectum, rectosigmoid junction, and distal sigmoid colon; long-segment type, with aganglionosis extending to the mid-sigmoid colon, descending colon, or transverse colon; and total colonic aganglionosis, with aganglionosis involving the entire colon (19).

### 2.2 Sample size

The sample size was based on the rate of excellent bowel preparation. Assuming *α* = 0.05, *β* = 0.10, *P*_1_ = 1.00, and *P*_2_ = 0.785 based on previous research (20), 43 participants were required per group. Allowing for a 10% attrition rate, 47 participants were recruited per group (total *n* = 94).

### 2.3 Randomization and blinding

An independent researcher generated the randomization sequence using Excel, and allocation was concealed in sealed envelopes. Participants were assigned to the intervention or control group at a 1:1 ratio. Blinding of intervention providers was not feasible because of the nature of the intervention. Participants, outcome assessors, and statisticians were blinded to group allocation.

### 2.4 Intervention

Both groups received standardized routine medical and nursing care from the same clinical team, and all healthcare professionals received standardized training in retrograde enema procedures.

#### 2.4.1 IM theory-based transitional health management

The intervention was developed through semi-structured interviews, literature review, expert consultation, and pilot testing.

An eight-member multidisciplinary team was established, comprising two pediatric surgery chief physicians, one head nurse, two senior surgical nurses, one resident physician, one clinical dietitian, and one psychological counselor. The team provided continuous support during hospitalization and home care. The intervention was developed according to the core processes of IM theory, including needs assessment, identification of intervention objectives, selection and development of intervention strategies, implementation, and evaluation (21).

Semi-structured interviews: Twelve primary caregivers were purposively recruited until data saturation. Braun and Clarke's thematic analysis was used (22), identifying three major needs: inadequate disease and enema knowledge, insufficient self-regulation, and inadequate social support. Accordingly, the intervention objectives were defined as improving disease knowledge, strengthening home-based self-management, and establishing a support system.

Literature review: Chinese and English databases were systematically searched from inception to November 2023. Two researchers independently screened the literature after duplicate removal using EndNote 20, and eight studies were included. Evidence was integrated with the identified needs to formulate the preliminary intervention.

Expert consultation: Ten experts from five tertiary hospitals participated in two rounds of consultation, including pediatric surgical nursing, clinical, nutrition, and psychology specialists. All questionnaires were validly returned (100% response rate), with an authority coefficient of 0.90 in both rounds. The intervention was revised according to expert recommendations.

Pilot study: Six children with HD and their caregivers participated in a 1-month pilot study. Recruitment and retention rates were both 100%, with no adverse events. Based on participant and provider feedback, a rotating on-call system and scheduled reminders were added to address missed enemas and delayed responses in the WeChat group. The final intervention protocol was established (Table 1).

**Table 1:** Final version of continuous health management program for children undergoing HD reflux enema.

Intervention Time	Intervention Content	Intervention Method	Intervention Duration and Frequency	Intervention Staff
After Initial Admission	1. Pre-intervention assessment: Proactively communicate with the primary caregiver of the child, initially assess general information and disease characteristic data, and understand the main current health problems of the child	Face-to-face communication	10–15 min, 2 times	Pediatric Surgeon, Primary Nurse
	2. Surgeon and Primary Nurse: Introduce knowledge of related diseases, explain the significance, methods and risks of reflux enema, and sign the Informed Consent Form	Face-to-face communication	10–15 min, 1 time	Pediatric Surgeon, Primary Nurse
	3. Dietitians: According to the results of nutritional risk screening at admission, provide routine introduction of dietary precautions during reflux enema for children with low risk; provide intervention by the dietitian for children with medium and high risk	Lecture	10–20 min, 1 time	Dietitian
	4. Primary Nurse: Lead the implementation of reflux enema. During the enema process, the primary nurse demonstrates the whole process of reflux enema, with emphasis on the timing selection, environment preparation, supplies preparation and pre-enema assessment of reflux enema; emphasize that the operation should be gentle and violence is strictly prohibited; the anal canal should pass through the stenotic segment; one person cooperates with clockwise abdominal massage; follow the principle that the output volume is ≥the input volume; pay attention to assessing the color, texture and odor of stool; the primary caregiver observes and learns	Demonstration and Lecture	15–30 min per time, 2 times in total	Primary Nurse
	5. Primary Nurse: Lead the implementation of reflux enema. During the enema process, the primary nurse demonstrates the whole process of reflux enema, with emphasis on risk identification: closely observe the child's complexion, mental status, breathing condition, etc. throughout the enema process. If the child is found to have pale complexion, poor mental status or meat-washing water-like liquid outflow, be alert to intestinal perforation, immediately stop bowel lavage and report to the doctor; the primary caregiver observes and learns	Demonstration and Lecture	5–10 min per time, 2 times in total	Primary Nurse
Three Days Before Initial Discharge	6. Pediatric Surgeon and Primary Nurse: The primary caregiver independently completes 3 times of reflux enema, with no principle errors in the whole enema operation process, and passes the joint assessment by the pediatric surgeon and the primary nurse	Operation Assessment	20–30 min, 3 times in total	Primary Caregiver, Pediatric Surgeon, Primary Nurse
	7. Pediatric Surgeon and Primary Nurse: Question the primary caregiver to confirm their awareness of the qualified criteria for reflux enema, the manifestations of reflux enema-related complications (intestinal perforation, enterocolitis, intestinal obstruction) and the emergency handling procedures for risks	Questioning	5–10 min, 1 time	Pediatric Surgeon, Primary Nurse
	8. Primary Nurse: Assess the completion of reflux enema by the primary caregiver of the child, and demonstrate and guide on the existing problems	Lecture and Demonstration	10 min, 3 times	Primary Nurse
	9. Psychological Counselor, Pediatric Surgeon and Primary Nurse: The primary nurse affirms the efforts and progress of the primary caregiver; the surgeon introduces successful cases of the same disease and gives encouragement; the psychological counselor helps them build confidence in long-term care after discharge	Psychological Support	20 min, 1 time	Psychological Counselor, Pediatric Surgeon, Primary Nurse
At Initial Discharge	10. Multidisciplinary Collaborative Team: Provide discharge education, with key education on adhering to medical advice for regular re-examination, insisting on daily reflux enema, nutrition management and follow-up	Individualized Guidance, Health Education	10–20 min, 1 time	Multidisciplinary Collaborative Nursing Team
	11. Members of the Multidisciplinary Collaborative Nursing Team: Guide the primary caregiver to join the WeChat Official Account and WeChat Group, modify the nickname as required, abide by the group rules, and do not invite others to join the group without permission; focus on introducing the functions and usage methods of the WeChat Group and WeChat Official Account			
	12. Primary Nurse strengthens the identification and response to emergency situations: Distribute the “Identification and Emergency Handling Checklist for Emergency Situations of Reflux Enema” and explain it			
	13. Dietitian: Set individualized nutrition intervention goals for the child; inform the individualized height and weight targets, to achieve and maintain ideal height and weight			
During Home Care Period	14. Primary Caregiver: Watch the reflux enema operation video for consolidated learning, and independently complete reflux enema daily	Mobile Video Education	5 min	Primary Caregiver
	15. Implementation of daily reflux enema: ① The primary nurse releases the enema task in the group at 08:00 every morning for supervision; ② The on-duty staff of the WeChat Group reminds the enema task at 12:00 and 18:00 every day respectively; ③ The on-duty staff counts the completion status through group check-in via WeChat Group at 18:30, and supervises the primary caregivers who do not check in via WeChat or phone, and follows up until the task is completed	WeChat Group, Phone Call	5–10 min per time, 4 times a day	Primary Nurse, On-duty Staff
	16. When the primary caregiver finds problems in disease care, nutrition management and psychology during home care, they can consult through the WeChat platform, and the members of the multidisciplinary team will answer according to their professional fields: ① Arrange medical staff to be on duty in the WeChat Group every day, set up first-line and second-line shifts, and reply to questions from 18:00 to 22:00; ② For emergency or sudden problems, refer to the “Identification and Emergency Handling Checklist for Emergency Situations of Reflux Enema”, and contact medical staff through the emergency contact channel if the problem cannot be solved	Individualized Guidance, WeChat Platform	Not fixed	Multidisciplinary Collaborative Nursing Team
	17. Pediatric Surgeon and Nurse: Regularly assess the implementation of reflux enema by the primary caregiver and their care ability for the child, and provide corresponding guidance according to the feedback results	One-to-one Communication	10–20 min (1 week and 1 month after discharge)	Pediatric Surgeon, Nurse
	18. Dietitian: Regularly push nutrition recipes for different age groups to help the primary caregiver with reasonable nutrition ratio and choose a reasonable diet pattern; when the primary caregiver finds nutrition-related problems, they can consult online through the WeChat Official Account or WeChat Group. The on-duty staff will feedback to the dietitian in time, and the dietitian will provide guidance and intervention in time	Individualized Guidance, WeChat Push	10–20 min per time, once every two weeks	Dietitian
	19. Psychological Counselor: Encourage the expression of confusion and negative emotions in the process of caring for the child, communicate with other primary caregivers in the group, and often affirm and encourage the progress of the primary caregiver; inform the ways to seek help, or provide guidance by the psychological counselor	Individualized Guidance, Peer Communication in WeChat Group	Not fixed	Psychological Counselor

#### 2.4.2 Implementation of the IM theory-based transitional health management program

The intervention group received the finalized IM theory-based transitional health management program in addition to routine care, delivered collaboratively by the multidisciplinary team. Before implementation, team members received standardized training, and caregivers were instructed on the use of WeChat. During the intervention, individualized remote guidance was provided through a WeChat public account and group, following the standardized intervention protocol (Table 1).

#### 2.4.3 Control group

The control group received routine care, including informed consent regarding the risks of retrograde enema and standardized enema training. Caregivers were required to independently perform three standardized procedures before discharge. Discharge education covered daily home-based retrograde enema, recognition of warning signs of intestinal perforation, and indications for emergency care. Follow-up visits were scheduled at 1 week and 1 month after discharge, with definitive surgery planned approximately 2–3 months later.

### 2.5 Data collection and outcome measures

#### 2.5.1 Primary outcomes

Preoperative bowel preparation was assessed using three indicators: (1) duration of preoperative enema, defined as the number of days from admission for definitive surgery to the final enema; (2) enema procedure time, defined as the time from initiation to clear effluent, or the time required to administer 150 mL/kg normal saline when clear effluent was not achieved; and (3) excellent intraoperative bowel preparation rate. Bowel cleanliness was independently assessed by two surgeons using a standardized grading system (23): excellent, clear mucosa without fecal fluid; good, clear mucosa with a small amount of fecal fluid; and poor, presence of a small amount of formed stool. The excellent rate was calculated as the proportion of patients rated excellent.

#### 2.5.2 Secondary outcomes

Enema-related complications: Enterocolitis, dermatitis, intestinal perforation, and intestinal obstruction diagnosed during the enema period were identified from electronic medical records at admission for definitive surgery.

Nutritional status and growth: Height and weight were measured using standardized procedures at initial admission and preoperatively. Height-for-age and weight-for-age *Z*-scores (HAZ and WAZ) were calculated (24), with HAZ or WAZ <−2 indicating growth retardation or underweight, respectively. Hemoglobin (Hb), albumin (ALB), and total protein (TP) levels were extracted from electronic medical records.

Caregiving ability: The Chinese version of the Family Caregiver Task Inventory (FCTI), revised by Lee (25), was used. The 25-item, five-domain scale uses a 3-point Likert scale (0–2), with higher scores indicating poorer caregiving ability. Its Cronbach's *α*was 0.913 (26). Assessments were performed at discharge and 1 week and 1 month after discharge using an online questionnaire.

Anxiety and depression: The Chinese versions of the Self-Rating Anxiety Scale (SAS) and Self-Rating Depression Scale (SDS) were used (27). Each scale contains 20 items rated on a 4-point Likert scale; standardized scores ≥50 indicate anxiety or depression. Cronbach's *α* coefficients were 0.841 and 0.91, respectively (28). Assessments were conducted at discharge and 1 week and 1 month after discharge.

### 2.6 Statistical analysis

Data were double-checked and analyzed using SPSS version 27.0. Continuous variables were assessed for normality using the Shapiro–Wilk test and presented as mean ± standard deviation or median (interquartile range), as appropriate. Between-group comparisons were performed using the independent-samples *t* test or Mann–Whitney *U* test, and within-group comparisons using the paired-samples *t* test where appropriate. Categorical variables were compared using the *χ*^2^ test, continuity-corrected *χ*^2^ test, or Fisher's exact test. Repeated-measures analysis of variance was used to assess changes in caregiving ability, anxiety, and depression over time. All tests were two-sided, with *P* < 0.05 considered statistically significant.

## 3 Results

### 3.1 Participant flow and baseline characteristics

A total of 94 participants were initially enrolled. During the study, five participants in the intervention group and six in the control group were lost to follow-up, leaving 83 participants for the final analysis (42 in the intervention group and 41 in the control group). No significant differences in baseline characteristics were observed between the groups (*P* > 0.05; Table 2).

**Table 2:** Comparison of baseline characteristics of children and their primary caregivers.

Population	Characteristics		Experimental group (*n* = 42)	Control group (*n* = 41)	*χ* ^2^ */Z/t*	*P*-value
			Median (P25,P75)/*n* (%)/Mean ± SD	Median (P25, P75)/*n* (%)/Mean ± SD		
Children	Age (months)		6.00 (5.00,6.25)	6.00 (5.00,9.00)	−0.895	0.371^b^
	Gender	Male	29 (69.05)	29 (70.73)	0.028	0.867^c^
		Female	13 (30.95)	12 (29.27)		
	HD type	Short-segment type	3 (7.14)	5 (12.19)	—	0.741^d^
		Common type	36 (85.72)	34 (82.93)		
		Long-segment type	3 (7.14)	2 (4.88)		
	Growth and development	HAZ	−0.69 ± 1.28	−1.18 ± 1.33	1.728	0.088^a^
		WAZ	−0.78 ± 1.28	−1.31 ± 1.58	1.676	0.098^a^
	Nutritional status	Hemoglobin (g/L)	106.31 ± 1.90	105.24 ± 2.17	0.37	0.712^a^
		Albumin (g/L)	38.10 ± 3.10	38.10 ± 4.25	−0.006	0.995^a^
		Total protein (g/L)	52.59 ± 5.17	52.50 ± 7.29	0.065	0.948^a^
Primary caregivers	Age (years)		32.00 (28.75,35.25)	30.00 (29.00,35.00)	−0.101	0.92^b^
	Gender	Male	12 (28.57)	8 (19.51)	0.931	0.335^c^
		Female	30 (71.43)	33 (80.49)		
	Residence	Urban	24 (57.14)	25 (60.98)	0.008	0.928^c^
		Rural	18 (42.86)	16 (39.02)		
	Relationship with the child	Parents	33 (78.57)	34 (82.93)	0.253	0.615^c^
		Grandparents or others	9 (21.43)	7 (17.07)		
	Educational level	High school and below	18 (42.86)	22 (53.66)	0.969	0.325^c^
		Above high school	24 (57.14)	19 (46.34)		
	Occupation	Farming	18 (42.86)	15 (36.59)	——	0.946^d^
		Office worker	14 (33.33)	15 (36.59)		
		Civil servant	3 (7.14)	5 (12.19)		
		Teacher	5 (11.90)	4 (9.76)		
		Others	2(4.76)	2(4.87)		

*χ*^2^, chi-square test; *Z*, normal approximation *Z* statistic for the Mann–Whitney *U* test; *t*, student's *t*-test; P25, 25th percentile; P75, 75th percentile; HAZ, height-for-age *Z*-score; WAZ, weight-for-age *Z*-score; SD, standard deviation.

a Independent two-sample *t*-test.

b Mann–Whitney *U* test.

c Chi-square test.

d Fisher's exact test.

### 3.2 Preoperative bowel preparation

The intervention group had a significantly shorter duration of preoperative enema and enema procedure time, as well as a higher rate of excellent intraoperative bowel cleanliness, than the control group (all *P* < 0.05; Table 3).

**Table 3:** Comparison of preoperative intestinal preparation effects between the two groups of children.

Group	Duration of preoperative enema (days)	Enema operation time (min)	Intraoperative intestinal cleanliness grade	
	Median (P25, P75)	Median (P25, P75)	Excellent *n* (%)	Non-excellent *n* (%)
Experimental group (*n* = 42)	4.50 (4.00,5.00)	20.00 (20.00,21.00)	39 (92.86）	3 (7.14）
Control group (*n* = 41)	7.00 (6.50,7.50)	37.00 (37.00,38.00)	29 (70.73）	12 (29.27)
*χ*^2^/*Z*	−7.927	−7.934	6.86	
*P-value*	<0.001^*^^,^^a^	<0.001^*^^,^^a^	0.009^*^^,^^b^	

*χ*^2^, chi-square test; *Z*, normal approximation *Z* statistic for the Mann–Whitney *U* test; P25, 25th percentile; P75, 75th percentile.

a Mann–Whitney *U* test.

b Chi-square test.

* *P* < 0.05.

### 3.3 Enema-related complications

The incidences of Hirschsprung-associated enterocolitis (HAEC) and overall complications were significantly lower in the intervention group than in the control group (*P* < 0.05). No significant between-group differences were observed in enema-related dermatitis or intestinal obstruction (*P* > 0.05), and no intestinal perforation occurred in either group (Table 4).

**Table 4:** Comparison of the incidence of complications between the two groups of children.

Group	Complications associated with reflux enema				Total cases of reflux enema-related complications *n* (%)
	Enema-associated dermatitis *n* (%)	Intestinal obstruction *n* (%)	Enterocolitis *n* (%)	Intestinal perforation *n* (%)	
Experimental group (*n* = 42)	1 (2.38)	0 (0.00)	2 (4.76)	0 (0.00)	3 (7.14)
Control group (*n* = 41)	2 (4.88)	1 (2.44)	9 (21.95)	0 (0.00)	12 (29.27)
*χ* ^2^	—	—	5.332	—	6.860
*P*-value	0.616^b^	0.494^b^	0.021^*^^,^^a^	—	0.009^*^^,^^a^

*χ*^2^, chi-square test.

a Chi-square test.

b Fisher's exact test.

* *P* < 0.05.

### 3.4 Growth, development, and nutritional status

#### 3.4.1 Between-group comparison before surgery

Before surgery, the intervention group had significantly higher HAZ, WAZ, Hb, ALB, and TP levels than the control group (all *P* < 0.05; Table 5).

**Table 5:** Comparison of growth, development and nutritional status between the two groups of children before surger**y.**

Group	Experimental group (*n* = 42)	Control group (*n* = 41)	*t*	*P*-value
	Mean ± SD	Mean ± SD		
WAZ	−0.43 ± 1.46	−1.42 ± 1.34	3.214	0.002*
HAZ	−0.42 ± 1.44	−1.40 ± 1.84	2.713	0.008*
Hemoglobin (g/L)	115.31 ± 12.30	109.24 ± 13.90	2.107	0.038*
Albumin (g/L)	43.10 ± 3.10	40.60 ± 4.25	3.064	0.003*
Total protein (g/L)	59.79 ± 5.17	55.90 ± 7.29	2.81	0.006*

*t*, student's *t*-test; SD, standard deviation; HAZ, height-for-age *Z*-score; WAZ, weight-for-age *Z*-score.

* *P* < 0.05.

#### 3.4.2 Within-group changes from initial diagnosis to surgery

HAZ and WAZ changed significantly from initial diagnosis to the preoperative assessment in both groups (both *P* < 0.001). HAZ and WAZ showed improving trends in the intervention group but declining trends in the control group, with significantly different trajectories between groups. Hb, ALB, and TP also increased significantly in both groups (all *P* < 0.001), with greater improvements in the intervention group. Detailed within-group comparisons are presented in Table 6 and Figure 1 illustrates the trajectories of growth and nutritional indicators.

**Table 6:** Comparison of self-changes in growth, development and nutritional status between the two groups of children at initial diagnosis and before surgery.

Group	Characteristics	Mean ± SD	MeanDiff (95% CI)	*t*	*P*-value
Experimental group (*n* = 42)	HAZ at initial diagnosis	−0.69 ± 1.28	−0.69 (−0.10, −0.38)	−4.55	<0.001^*^^,^^a^
	HAZ before surgery	−0.42 ± 1.44			
	WAZ at initial diagnosis	−0.78 ± 1.28	−0.91 (−1.13, −0.68)	−8.03	<0.001^*^^,^^a^
	WAZ before surgery	−0.43 ± 1.46			
	Hemoglobin at initial diagnosis	106.66 ± 12.06	8.64 (8.23, 9.06)	41.79	<0.001^*^^,^^a^
	Hemoglobin before surgery	115.31 ± 12.30			
	Albumin at initial diagnosis	37.62 ± 4.48	−5.47 (−6.39, −4.55)	−12.03	<0.001^*^^,^^a^
	Albumin before surgery	43.10 ± 3.10			
	Total protein at initial diagnosis	52.59 ± 5.13	−7.20 (−7.34, −7.05)	−100.18	<0.001^*^^,^^a^
	Total protein before surgery	59.79 ± 5.17			
Control group (*n* = 41)	HAZ at initial diagnosis	−1.18 ± 1.33	−0.38 (−0.67, −0.08)	−2.58	0.014^*^^,^^a^
	HAZ before surgery	−1.40 ± 1.84			
	WAZ at initial diagnosis	−1.31 ± 1.58	−0.75 (−0.90, −0.59)	−9.92	<0.001^*^^,^^a^
	WAZ before surgery	−1.42 ± 1.34			
	Hemoglobin at initial diagnosis	105.29 ± 13.61	3.95 (3.64, 4.27)	25.33	<0.001^*^^,^^a^
	Hemoglobin before surgery	109.24 ± 13.90			
	Albumin at initial diagnosis	38.10 ± 4.13	−2.44 (−2.57, −2.32)	−39.84	<0.001^*^^,^^a^
	Albumin before surgery	40.60 ± 4.25			
	Total protein at initial diagnosis	52.37 ± 7.21	−3.52 (−3.64, −3.40)	−59.08	<0.001^*^^,^^a^
	Total protein before surgery	55.90 ± 7.29			

*t*, student's *t*-test; SD, standard deviation; MeanDiff, mean difference; 95% CI, 95% confidence interval; HAZ, height-for-age *Z*-score; WAZ, weight-for-age *Z*-score.

a Paired-samples t-test;

* *P* < 0.05.

**Figure 1:**
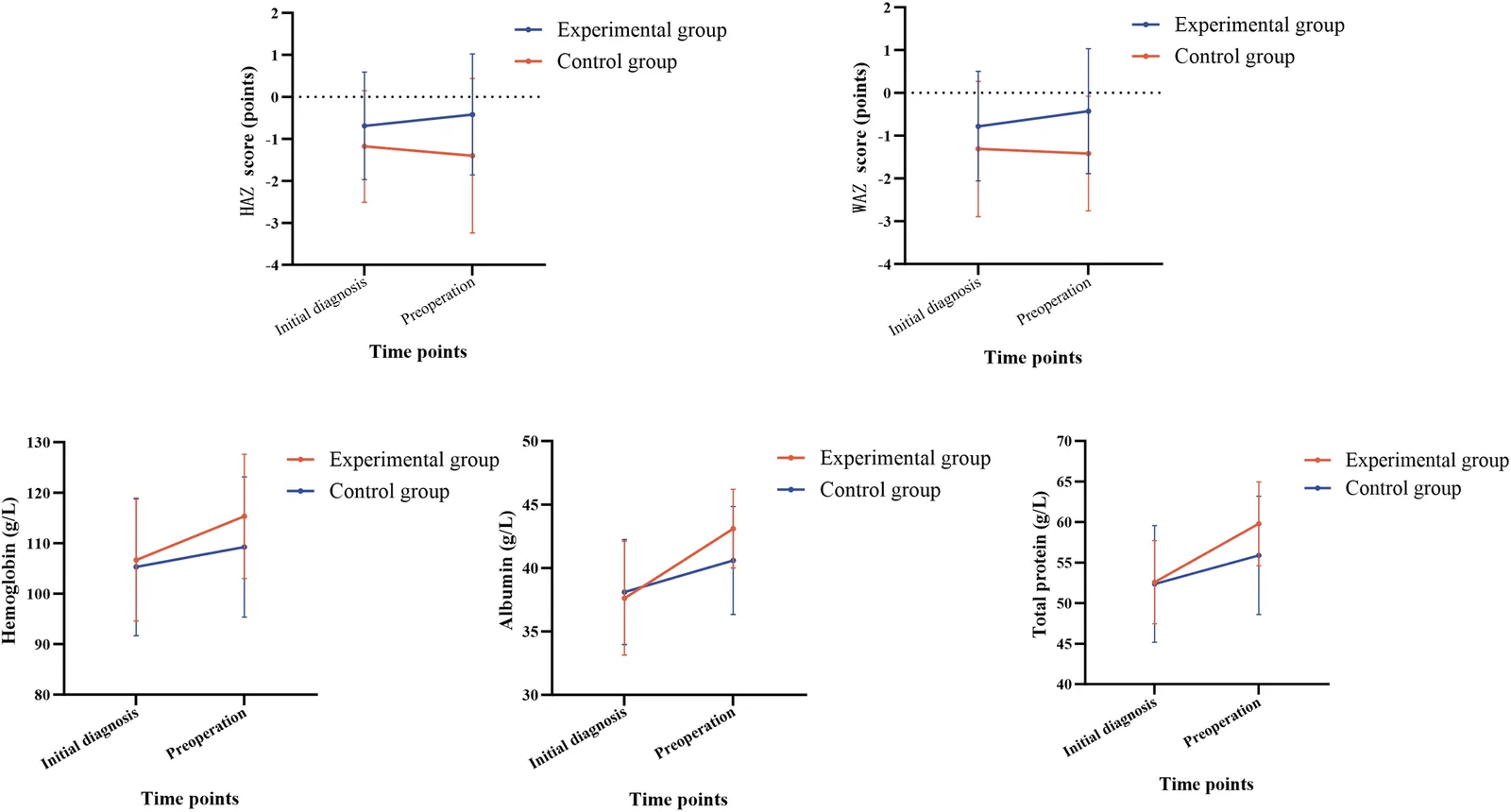
Trend chart of self-changes in growth, development and nutritional status of two groups of children at initial diagnosis and before surgery.

### 3.5 Caregiving ability

Repeated-measures ANOVA showed significant effects of group, time, and group-by-time interaction (all *P* < 0.001). There was no significant between-group difference at discharge (*P* > 0.05), whereas FCTI scores were significantly lower in the intervention group at 1 week and 1 month after discharge, indicating better caregiving ability. The between-group difference increased over time (Table 7 and Figure 2).

**Table 7:** Results of repeated measures analysis of variance for caregivers’ caregiving ability (FCTI).

Characteristics	At discharge	1 week after discharge	1 month after discharge	Results of repeated-measures *F*-test		
	Mean ± SD	Mean ± SD	Mean ± SD	F Partial	*P*-value	Partial *η*^2^
Control group	34.93 ± 1.35	31.85 ± 1.90	30.59 ± 1.72	—	—	—
Experimental group	34.81 ± 1.35	30.12 ± 1.31	26.55 ± 2.01	—	—	—
Main effect of Group	—	—	—	90.802	<0.001*	0.529
Main effect of Time	—	—	—	313.671	<0.001*	0.795
Group*Time interaction	—	—	—	30.120	<0.001*	0.271

SD, standard deviation; *F* partial, partial *F* statistic; Partial *η*^2^, partial eta-squared.

* *P* < 0.05.

**Figure 2:**
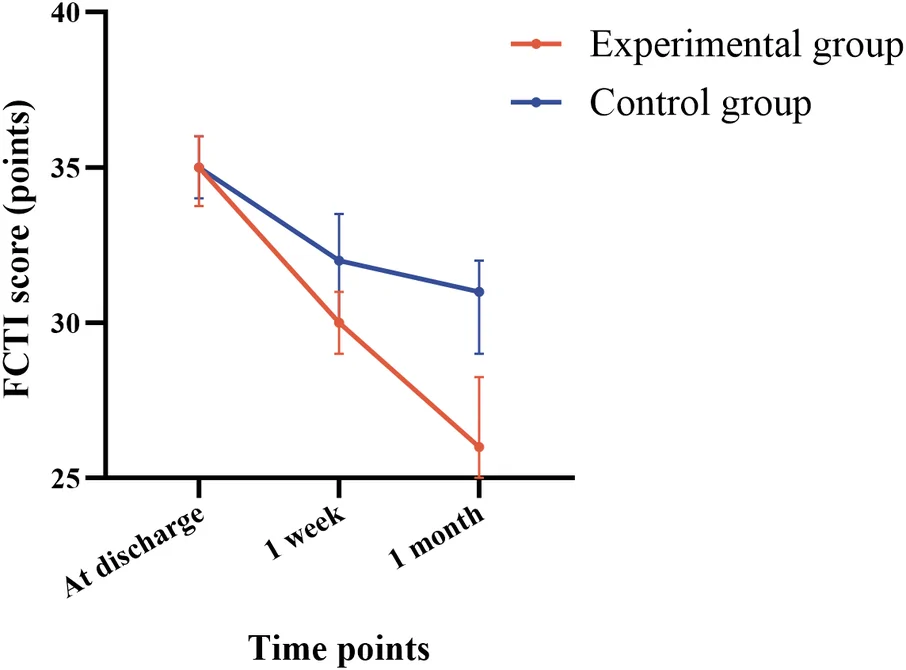
Line graph showing the profile of repeated measures of caregivers’ caregiving ability. Family Caregiver Task Inventory (FCTI) scores were compared between the intervention and control groups at discharge, 1 week, and 1 month after discharge. Error bars indicate standard deviations (SDs).

### 3.6 Anxiety

Significant effects of group, time, and group-by-time interaction were observed for SAS scores (all *P* < 0.001). No between-group difference was found at discharge (*P* > 0.05). At 1 week and 1 month after discharge, significant group and time effects were observed (both *P* < 0.001). SAS scores decreased over time in both groups, with a greater reduction in the intervention group, indicating greater improvement in anxiety (Table 8 and Figure 3).

**Table 8:** Results of repeated measures analysis of variance for caregivers’ anxiety scores (SAS).

Characteristics	At discharge	1 week after discharge	1 month after discharge	Results of repeated-measures *F*-test		
	Mean ± SD	Mean ± SD	Mean ± SD	F Partial	*P*-value	Partial *η*^2^
Control group	69.83 ± 3.29	59.34 ± 3.45	52.93 ± 3.76	—	—	—
Experimental group	70.67 ± 2.76	57.36 ± 3.46	48.69 ± 3.36	—	—	—
Main effect of Group	—	—	—	19.401	<0.001*	0.193
Main effect of Time	—	—	—	678.581	<0.001*	0.893
Group*Time interaction	—	—	—	11.414	<0.001*	0.124

SD, standard deviation; *F* Partial, partial *F* statistic; Partial *η*^2^, partial eta-squared.

* *P* < 0.05.

**Figure 3:**
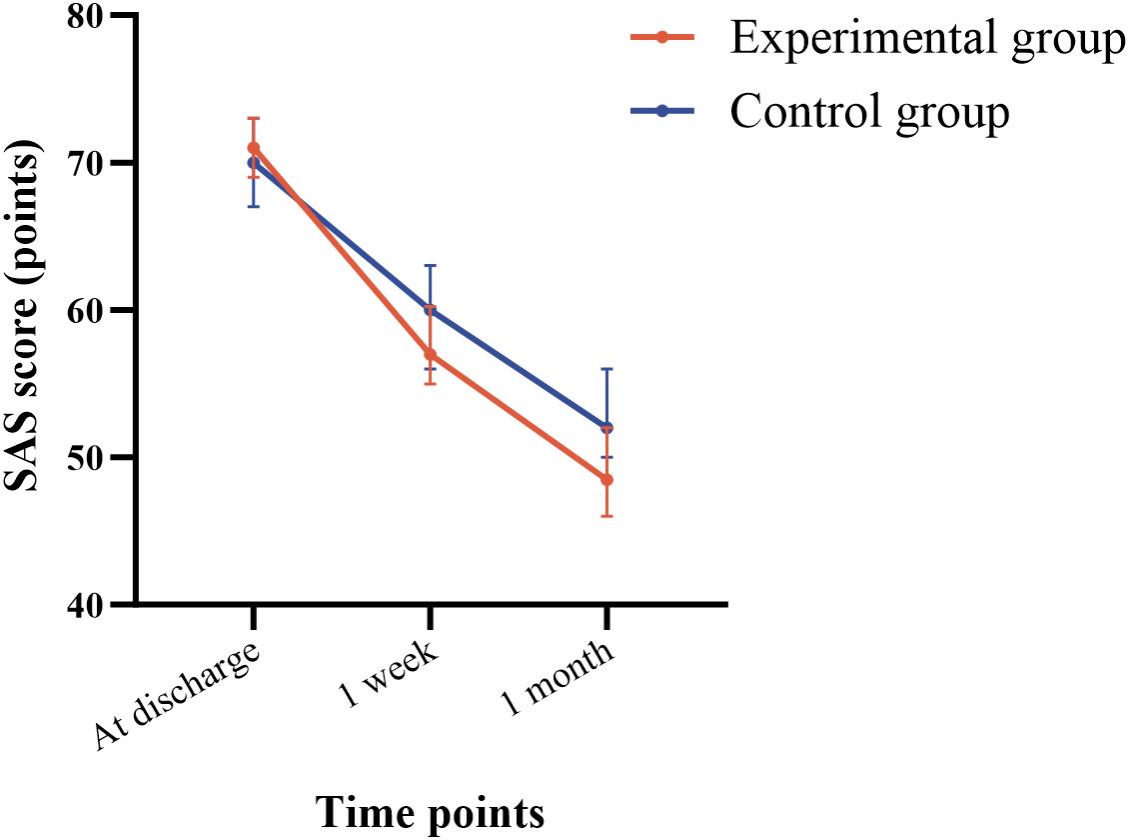
Line graph showing the profile of repeated measures of primary caregivers’ anxiety. Self-Rating Anxiety Scale (SAS) scores were compared between the intervention and control groups at discharge, 1 week, and 1 month after discharge. Error bars indicate standard deviations (SDs).

### 3.7 Depression

Repeated-measures ANOVA showed significant effects of group, time, and group-by-time interaction for SDS scores (all *P* < 0.001). No significant between-group difference was observed at discharge (*P* > 0.05). At 1 week and 1 month after discharge, significant group and time effects were observed (both *P* < 0.001). SDS scores decreased progressively in both groups, with a greater reduction in the intervention group, indicating greater improvement in depressive symptoms (Table 9 and Figure 4).

**Table 9:** Results of repeated measures analysis of variance for caregivers’ depression scores (SDS).

Characteristics	At discharge	1 week after discharge	1 month after discharge	Results of repeated-measures *F*-test		
	Mean ± SD	Mean ± SD	Mean ± SD	F Partial	*P*-value	Partial *η*^2^
Control group	60.05 ± 3.29	56.46 ± 3.70	43.68 ± 4.59	—	—	—
Experimental group	60.21 ± 3.18	51.74 ± 3.61	36.71 ± 3.78	—	—	—
Main effect of Group	—	—	—	71.408	<0.001*	0.469
Main effect of Time	—	—	—	607.056	<0.001*	0.882
Group*Time interaction	—	—	—	19.328	<0.001*	0.193

SD, standard deviation; *F* Partial, partial *F* statistic; Partial *η*^2^, partial eta-squared.

* *P* < 0.05.

**Figure 4:**
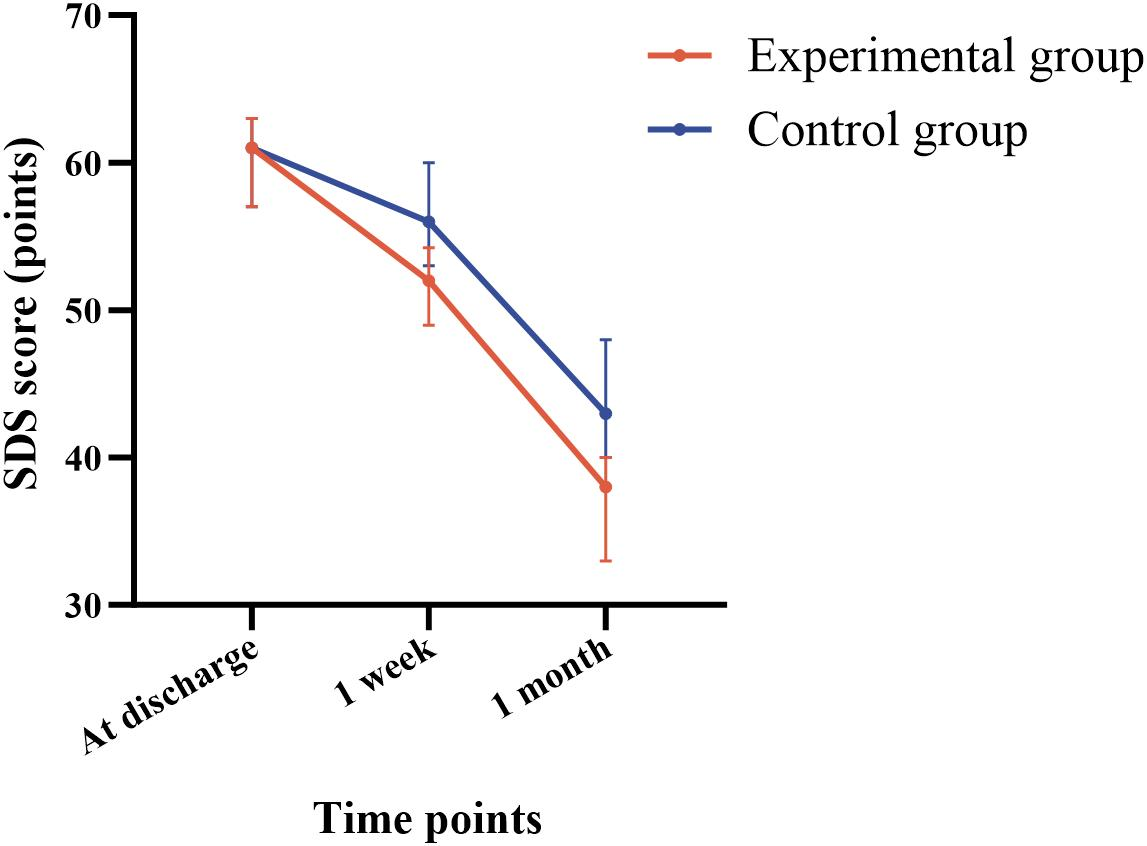
Line graph showing the profile of repeated measures of primary caregivers’ depression. Self-Rating Depression Scale (SDS) scores were compared between the intervention and control groups at discharge, 1 week, and 1 month after discharge. Error bars indicate standard deviations (SDs).

## 4 Discussion

### 4.1 Scientific rationale and clinical feasibility of the intervention

This study developed an IM theory-based transitional health management program tailored to the clinical characteristics of children with HD and the challenges of home-based care. The program integrated caregiver needs identified through semi-structured interviews with evidence from the literature and expert consultation. The multidisciplinary expert panel included specialists in pediatric surgery, nursing, nutrition, and psychology, and the authority coefficient was 0.90 in both consultation rounds, supporting the credibility of the development process. By addressing children and caregivers as an integrated care unit, the program incorporated procedural guidance, nutritional management, and psychological support throughout the care process. Pilot testing further confirmed its feasibility and practical applicability.

### 4.2 Effects and potential advantages of the intervention

The intervention significantly shortened the duration of preoperative enemas and individual enema procedures and improved intraoperative bowel cleanliness. These findings are consistent with previous studies (22, 29), suggesting that IM-based transitional health management may improve the efficiency and quality of home-based bowel preparation. Conventional discharge education may be insufficient because caregivers may forget procedural details after limited training. In contrast, the present intervention provided standardized instructional videos and real-time professional guidance through WeChat, allowing caregivers to obtain timely feedback and correction. This approach extends professional nursing support into the home and may overcome limitations of conventional discharge education in pediatric surgical care.

The intervention also reduced the incidence of HAEC and overall complications, consistent with previous findings (30). HAEC is a major complication of HD and is associated with bacterial overgrowth and impaired intestinal mucosal integrity (4). Early recognition of warning signs and effective bowel decompression are therefore important preventive measures. The multidisciplinary team used the WeChat platform to facilitate timely identification of early symptoms and provide individualized guidance, while dietary education supported appropriate nutritional and intestinal management. No significant differences were observed in dermatitis or intestinal obstruction, possibly because of the low event rates and limited sample size. No intestinal perforation occurred, and the effect of the intervention on mechanical complications requires confirmation in larger studies. Because the timing and severity of HAEC varied among participants, future studies should consider stratified analyses according to HAEC history.

The intervention was also associated with better preoperative growth and nutritional status. HD-related mechanical obstruction can impair appetite and nutrient absorption, contributing to growth retardation and anemia, while adequate nutritional status is essential for surgery and recovery (31–33). In the present study, growth indicators improved from initial diagnosis to surgery in the intervention group, while biochemical nutritional indicators improved in both groups, with greater improvements in the intervention group. Through the WeChat platform, caregivers received timely feeding advice, guidance on recognizing inadequate bowel evacuation, individualized dietary recommendations based on weight and stool characteristics, and regular nutrition education. These components may have promoted active nutritional management during home care.

Caregiving ability improved progressively after discharge, with a greater improvement in the intervention group. This finding is consistent with previous studies (34, 35) and suggests a cumulative effect of continued support. Unlike conventional care, in which professional support often decreases rapidly after discharge, the present intervention provided ongoing video demonstrations, real-time consultation, and peer communication through the online platform. Such continuous bidirectional support may reduce uncertainty, strengthen caregiving skills, and enhance caregiver self-efficacy.

Caregivers of children with HD may experience substantial psychological burden, and anxiety and depression may further interfere with caregiving quality (36). In this study, both anxiety and depression decreased over time, with greater improvements in the intervention group, consistent with previous reports (37, 38). Improved caregiving skills may reduce uncertainty and psychological stress, while timely online consultation and standardized educational materials provide additional reassurance. Peer communication through the WeChat group may also provide emotional support. The progressively greater effects over time suggest that this model may be particularly suitable for children requiring prolonged home-based enema management.

### 4.3 Limitations

Several limitations should be considered. First, this single-center study included a relatively small sample from one tertiary pediatric hospital in Fujian Province, which may limit the generalizability of the findings. Multicenter studies with larger samples are needed to validate the intervention. Second, only short-term outcomes were assessed, including preoperative outcomes and follow-up at 1 week and 1 month after discharge; therefore, the long-term sustainability of the intervention remains uncertain. Third, although no significant between-group differences were observed in baseline HAZ and WAZ, the two groups showed different trajectories of growth during the study period. These divergent trends may have been influenced by multiple factors, and the relatively small sample size limits the ability to fully account for potential confounding. Therefore, the observed effects of the intervention on growth outcomes should be interpreted cautiously. Future studies with larger samples and adjusted analyses are warranted to further confirm these findings. Finally, although the intervention group had a lower incidence of HAEC than the control group, HAEC is influenced by multiple factors, including disease severity, aganglionic segment length, intestinal dysmotility, and the effectiveness of bowel decompression. Thus, its independent contribution to the observed outcomes could not be fully determined. Further multicenter studies with larger samples and subgroup analyses are warranted to confirm these findings and clarify the relationship between HAEC and transitional health management.

## 5 Conclusion

An IM theory-based transitional health management program integrating remote guidance, multidisciplinary support, and caregiver participation improved preoperative bowel preparation, reduced HAEC and overall enema-related complications, and improved growth and nutritional outcomes in children with HD receiving home-based retrograde enema. The program also enhanced caregivers’ caregiving ability and alleviated anxiety and depression. This approach may provide a practical framework for transitional care of children with HD and their caregivers.

## Data Availability

The original contributions presented in the study are included in the article/Supplementary Material, further inquiries can be directed to the corresponding author.
